# The Bradykinin B2 Receptor Agonist (NG291) Causes Rapid Onset of Transient Blood–Brain Barrier Disruption Without Evidence of Early Brain Injury

**DOI:** 10.3389/fnins.2021.791709

**Published:** 2021-12-15

**Authors:** Sergio R. Rodríguez-Massó, Michelle A. Erickson, William A. Banks, Henning Ulrich, Antonio Henrique Martins

**Affiliations:** ^1^Department of Pharmacology and Toxicology, University of Puerto Rico Medical Sciences Campus, San Juan, PR, United States; ^2^Geriatric Research Education and Clinical Center, Veterans Affairs Puget Sound Health Care System, Seattle, WA, United States; ^3^Division of Gerontology and Geriatric Medicine, Department of Medicine, School of Medicine, University of Washington, Seattle, WA, United States; ^4^Department of Biochemistry, Institute of Chemistry, University of São Paulo, São Paulo, Brazil

**Keywords:** bradykinin B2 receptor, blood–brain barrier, bradykinin, peptide, drug delivery

## Abstract

**Background:** The blood–brain barrier (BBB) describes the brain’s highly specialized capillaries, which form a dynamic interface that maintains central nervous system (CNS) homeostasis. The BBB supports the CNS, in part, by preventing the entry of potentially harmful circulating molecules into the brain. However, this specialized function is challenging for the development of CNS therapeutics. Several strategies to facilitate drug delivery into the brain parenchyma *via* disruption of the BBB have been proposed. Bradykinin has proven effective in disrupting mechanisms across the blood–tumor barrier. Unfortunately, bradykinin has limited therapeutic value because of its short half-life and the undesirable biological activity elicited by its active metabolites.

**Objective:** To evaluate NG291, a stable bradykinin analog, with selective agonist activity on the bradykinin-B2 receptor and its ability to disrupt the BBB transiently.

**Methods:** Sprague Dawley rats and CD-1 mice were subjected to NG291 treatment (either 50 or 100 μg/kg, intravenously). Time and dose-dependent BBB disruption were evaluated by histological analysis of Evans blue (EB) extravasation. Transcellular and paracellular BBB leakage were assessed by infiltration of ^99m^Tc-albumin (66.5 KDa) and ^14^C-sucrose (340 Da) radiolabeled probes into the brains of CD-1 mice treated with NG291. NG291 influence on P-glycoprotein (P-gp) efflux pump activity was evaluated by quantifying the brain accumulation of ^3^H-verapamil, a known P-gp substrate, in CD-1 mice.

**Results:** NG291-mediated BBB disruption was localized, dose-dependent, and reversible as measured by EB extravasation. ^99m^Tc-albumin leakage was significantly increased by 50 μg/kg of NG291, whereas 100 μg/kg of NG291 significantly augmented both ^14^C-sucrose and ^99m^Tc-albumin leakage. NG291 enhanced P-gp efflux transporter activity and was unable to increase brain uptake of the P-gp substrate pralidoxime. NG291 did not evoke significant short-term neurotoxicity, as it did not increase brain water content, the number of Fluoro-Jade C positive cells, or astrocyte activation.

**Conclusion:** Our findings strongly suggest that NG291 increases BBB permeability by two different mechanisms in a dose-dependent manner and increases P-gp efflux transport. This increased permeability may facilitate the penetration into the brain of therapeutic candidates that are not P-gp substrates.

## Introduction

The blood–brain barrier (BBB) is often a primary factor that affects the viability of novel central nervous system (CNS) therapeutics. Lipinski’s rule of 5 has served in the past as a set of parameters that screen therapeutic candidates that can reach fluid compartments in the CNS (Lipinski et al., 2001; Bauer et al., 2014). Small molecules with a molecular weight under 500 Da, less than 5 hydrogen bond donors, less than 10 hydrogen bond acceptors, and a calculated logP less than 5 are expected to have a greater capacity to reach the CNS. Medicinal chemistry has been employed to increase BBB permeation of therapeutics, but this often increases penetration across all biological membranes, substantially decreasing the availability of the drug for the BBB (Begley, 2004; Pardridge, 2007). Strategies involving administration of hyperosmotic solutions like mannitol or high-frequency ultrasound mediate BBB disruption by disengaging the tight junctions, which results in increased paracellular transport of smaller water-soluble compounds (Han et al., 2017). Bradykinin (BK; *H-Arg^1^-Pro^2^-Pro^3^-Gly^4^-Phe^5^-Ser^6^-Pro^7^-Phe^8^-Arg^9^-OH*) is an endogenous vasoactive peptide that can reversibly increase the permeability of the blood–brain or blood–tumor barriers (Sanovich et al., 1995; Bartus et al., 1996a; Bartus, 1999; Borlongan and Emerich, 2003; Liu et al., 2015). BK mediates its effect through the constitutively expressed bradykinin B2 receptor (BKB2R), while its metabolite des-Arg^9^-BK, with a longer half-life (above 300 s), is a bradykinin B1 receptor (BKB1R) agonist (Bhoola et al., 1992; Zhang et al., 2021). Activation of BKB1R leads to increased self-expression. Increased levels of BKB1R are associated with several disease states (Golias et al., 2007). During epileptogenesis, BKB1R stimulation was associated with increased hippocampal cell death and mossy fiber sprouting, while BKB2R agonism has the opposite effect (Adolfo Argañaraz et al., 2004). With traumatic brain injury, increased BK levels have been associated with the development of vasogenic edema. While one study found a 50% decrease in brain water content in BKB2R (−/−) transgenic mice compared to wild-type animals (Trabold et al., 2010), another group found BKB1R inhibition rather than BKB2R inhibition reduced brain edema (Raslan et al., 2010). BKB1R inhibition is associated with decreased axonal injury, astrogliosis, and microglial migration (Ifuku et al., 2007; Albert-Weissenberger et al., 2012). Unlike BKB1R, BKB2R receptor internalization occurs following prolonged stimulation of its agonist (Haasemann et al., 1998). Therefore, a physiologically stable peptide that selectively stimulates BKB2R may promote a transient increase in BBB permeability that could be of therapeutic value.

Activation of BKB2R leads to the hydrolysis of phosphatidylinositol and increased intracellular calcium concentrations (Bartus et al., 1996b). BKB2R signaling events are mediated by mitogen-activated protein kinase (MAPK), cytosolic phospholipases A2, guanylate cyclase, and endothelial nitric oxide synthase (eNOS) pathways (Moreau et al., 2005). BKB2R-mediated functions have been described for the CNS, such as neurotransmission, neuroprotection, neurogenesis, inflammation control, and BBB permeability (Martins et al., 2012; Negraes et al., 2015; Pillat et al., 2016; Ji et al., 2017; Zhang et al., 2021). The literature suggests that BKB2R-mediated BBB disruption occurs *via* increased paracellular transport (Emerich et al., 2001; Saaber et al., 2014). BKB2R-mediated BBB disruption has been shown repeatedly with electron microscopy images of lanthanum accumulation detected between tight junctions with little to no lanthanum observed in endosomes (Sanovich et al., 1995; Emerich et al., 2001; Borlongan and Emerich, 2003; Saaber et al., 2014). Electron microscopy studies suggest that BKB2R-mediated BBB disruption could facilitate the paracellular delivery of small (under 500 Da) water-soluble compounds, such as pralidoxime (2-PAM; 137 Da) (Ferchmin et al., 2015). However, later studies have found that lanthanum can prevent intracellular calcium mobilization (dos Remedios, 1981; Korc and Schöni, 1987; Jan et al., 1998). Previous studies using lanthanum for confirming BKB2R-mediated paracellular transport may have suffered from limited signaling events, as otherwise increased transcellular transport would have been detected. A growing number of studies have found BKB2R-mediated transcellular transport across the BBB (Riethmüller et al., 2006; Jungmann et al., 2008; Bawolak et al., 2011). Selective BKB2R agonists are promising for delivering chemotherapeutics, such as carboplatin, loperamide, and cyclosporin-A, across the blood–brain and blood–tumor barrier (Borlongan and Emerich, 2003; Côté et al., 2012). The BK analog NG291 ([*Hyp*^3^, *Thi*^5^, *^N^Chg*^7^, *Thi*^8^]-BK) exhibits a high affinity toward human BKB2R (Savard et al., 2013). Cα trisubstituted unnatural residues (*Thi*^5,8^ and *^N^Chg*^7^) prevent NG291 cleavage by angiotensin-converting enzyme and carboxypeptidases (Savard et al., 2013).

As discussed above, BKB2R-mediated BBB disruption is promising, and further studies are encouraged. However, little work has been done to evaluate the risks involved with BKB2R-mediated BBB disruption. While BKB2R receptor internalization has been reported, little is known about how this effect translates into BBB permeability. In this study, we evaluate the effect of NG291 has on a normal BBB and clarify the modulation of transport routes [i.e., paracellular transport, transcellular transport, P-glycoprotein (P-gp) efflux transport activity]. Several studies were conducted to assess if NG291 may cause vasogenic edema, activate astrocytes; elicit axonal demyelination, or contribute to neurodegeneration. Results from this work provided a greater understanding of the effect of NG291 on a healthy normal BBB and delineated therapeutic classes that would benefit from NG291 facilitated transport across the CNS.

## Materials and Methods

### ADMET Property Prediction

The pharmacodynamics, pharmacokinetics, and toxicological profiles of bradykinin and NG291 were computed using the pkCSM tool^1^ as described by Pires et al. (2015) and Oladele et al. (2021). The canonical SMILE molecular structures of the compounds used in the studies were obtained from the JSME editor.^2^ The results obtained by running the pkCSM tool on bradykinin and NG291 are summarized in Table 1.

**TABLE 1:** Predicted pharmacokinetic properties of bradykinin and NG291.

Property	Descriptor	Bradykinin	NG291
Molecular property	Molecular weight	1060.228	1130.336
Molecular property	#Rotatable bonds	27	29
Molecular property	#Acceptors	13	16
Molecular property	#Donors	12	15
Molecular property	Surface areas	439.9	459.07
Absorption	Water solubility (mol/L)	0.001282331	0.001282331
Absorption	Caco-2 permeability (Papp in 10^–6^ cm/s)	0.287078058	0.235504928
Absorption	P-glycoprotein substrate (yes/no)	Yes	Yes
Absorption	P-glycoprotein I inhibitor (yes/no)	No	No
Absorption	P-glycoprotein II inhibitor (yes/no)	No	No
Distribution	VDss (human) (L/kg)	0.208449088	0.086896043
Distribution	Fraction unbound (human) (Fu)	0.427	0.462
Distribution	BBB permeability (log BB)	−2.137	−2.592
Distribution	CNS permeability (log PS)	−6.197	−6.465
Metabolism	CYP2D6 substrate (yes/no)	No	No
Metabolism	CYP3A4 substrate (yes/no)	Yes	Yes
Metabolism	CYP1A2 inhibitor (yes/no)	No	No
Metabolism	CYP2C19 inhibitor (yes/no)	No	No
Metabolism	CYP2C9 inhibitor (yes/no)	No	No
Metabolism	CYP2D6 inhibitor (yes/no)	No	No
Metabolism	CYP3A4 inhibitor (yes/no)	No	No
Excretion	Total clearance (ml/min/kg)	0.571478637	2.747894153
Toxicity	AMES toxicity (yes/no)	No	No
Toxicity	hERG I inhibitor (yes/no)	No	No
Toxicity	hERG II inhibitor (yes/no)	No	Yes

*The chemical structure of bradykinin and NG291 were used to develop a coded sequence by entering their chemical structure in SMILES format. Entering the SMILES sequence provided us with the ADMET properties.*

### Animals

All animals were housed and handled following protocols approved by Universidad Central del Caribe or the Veterans Affairs Puget Sound Health Care System’s Institutional Animal Care and Use Committee (IACUC). All experiments were conducted in accordance with the National Institutes of Health Guide for the Care and Use of Laboratory Animals. The animals used in this work were: Sprague Dawley (SD) rats (104 males and 57 females with 8–9 weeks of age from Universidad Central del Caribe colony weighing 250–300 g) and CD-1 mice (154 males 8–9 weeks old from Jackson Laboratories weighing 30–40 g) with food and water *ad libitum* with 12 h day/night cycles. Animal numbers used were based on calculations for typical studies to detect a 10% difference in the mean values (alpha = 0.05) with a power above 80%.

### Assessing Evans Blue Extravasation Following NG291 Administration

NG291 (Savard et al., 2013) was purchased from MediLumine.^3^ SD rats were anesthetized with a mixture of 1.5% isoflurane/70% nitrous oxide/30% oxygen. The anesthetized animals were injected (i.v.) with either saline, 50 or 100 μg/kg of NG291. The injected solution circulated for 1–8 h before an IV injection of 4 ml/kg 2% Evans blue (Millipore) solution (EB) dissolved in 0.9% saline. Sixteen hours after EB injection, the animals were submitted to transcardial perfusion with saline 0.9%. A blunt needle was introduced through the left ventricle and held in position when the needle head was visible in the aorta. The right atrium was punctured as an outlet for drainage of the perfusion fluid. Discoloration of the liver was used to confirm successful perfusion. After the perfusion, the brains were coronally sectioned using a stainless-steel brain matrix (Stoelting). Coronal sections (2 mm thick) were placed on a glass mount which allowed brains to be scanned dorsally and ventrally using an Epson Perfection V39 Scanner. The percent area of EB detected in the brain in proportion to the total brain area was calculated using ImageJ software (United States National Institutes of Health, Bethesda, MD, United States^4^).

Additionally, saline perfused brains were also coronally sectioned at 20 μm thickness using a Leica CM1850 microtome. EB (620 nm excitation/680 nm emission) extravasation to brain parenchyma was detected with a Keyence BZ-X800 fluorescence microscope after mounting slides with mounting media containing DAPI (Saria and Lundberg, 1983; Wang and Lai, 2015).

### Radiolabeled Tracer Preparation

The radiolabeled probes used to detect differences in transcytosis in NG291-injected animals and controls were prepared by dissolving 2 mg of bovine serum albumin (BSA) (Sigma A-7030) and 250 μg of Stannous tartrate (MP Biomedicals: ICN221948) in 1 ml of deionized water and adjusting to a pH of 3.0. Subsequently, one millicurie of ^99m^Tc-NaOH_4_ (GE Healthcare, Piscataway, NJ, United States) was added to the solution and incubated for 20 min at room temperature. Then, the ^99m^Tc-albumin was purified using a G-10 Sephadex (GE Healthcare) column in 0.1 ml fractions of phosphate buffer (0.25 M). The reaction efficiency was evaluated by acid precipitation with 30% trichloroacetic acid. Reaction efficiencies under 87% were discarded (Logsdon et al., 2018). The prepared ^99m^Tc-albumin [10 × 10^6^ counts per minute (cpm)/mouse] was combined with 0.75 μCi/mouse (1.665 × 10^6^ dpm/mouse) of C^14^-sucrose (Perkin Elmer, Waltman, MA, United States) and dissolved in lactated Ringer’s solution containing 1% BSA (at the final volume of 0.2 ml/mouse). Either 50 or 100 μg/kg of NG291 were administered to assess changes in radiolabeled probe accumulation in brain parenchyma.

A tritiated 2-PAM probe was used to verify whether 2-PAM would access the brain parenchyma following NG291 administration. To prepare the solution, 0.1 μCi/mouse of ^3^H-pralidoxime (^3^H-2-PAM, American Radiolabeled Chemicals, ART 2162-50 μCi; specific activity: 10 Ci/mmol) was constituted with 100 μg/kg of NG291 in lactated Ringer’s solution containing 1% BSA (final volume of 0.2 ml/mouse).

### Evaluating NG291 Mediated Blood–Brain Barrier Permeability With Radiolabeled Tracers

CD-1 Mice were anesthetized with urethane (4 g/kg; 0.2 ml i.p.). Once the animals had been sedated, the jugular veins were exposed, and intravenous co-injection of NG291 with the radiolabeled tracers was administrated. The injectate circulated for 15 min then the blood was collected from the severed inferior carotid artery. Immediately, transcardial perfusion was performed with 20 ml of Lactated Ringer’s solution. The brain was harvested and weighed before the counting of retained radioactivity. Blood was centrifuged for 10 min at 3500 × *g* and 4°C, and 20 μl of serum was collected for counting. At the end of the study, 20 μl of the injectate was collected as an “injection check,” which is used as a standard to determine the amount of tracer present in the provided injectate. Samples were counted in a gamma counter for 3 min to determine cpm per tissue weight for the brain samples or per 20 μl in the case of serum samples and injection check. Solvable™ (1.5 ml, Perkin Elmer, catalog 6NE9100) was then added to each sample tube and sealed. Samples were then stored until the brain tissue was fully dissolved and technetium decayed. After dissolution, they were counted in a beta counter to quantify ^14^C or ^3^H concentrations in terms of dpm/g for brain samples or dpm/μl for serum samples and injection checks. Radiolabeled probe accumulation in brain parenchyma is presented in terms of brain to serum ratios (μl/g).

### F-Actin Expression on Brain Treated With NG291

Sprague Dawley rat treated with saline (control), acute NG291 (100 μg/kg NG291 3 days prior to saline perfusion), and chronic NG291 (100 μg/kg NG291 per day for 3 days for three doses) with brain perfusion conducted 1 h after the final dose was sectioned 20 μm thick. Brain sections were stained for F-actin (phalloidin, Alexa Fluor™ 488, green) and nuclei (DAPI, blue). Stained slides were observed under a Keyence BZ-X800 fluorescence microscope and imaged.

### Baseline Pralidoxime Kinetics

CD-1 mice were anesthetized with urethane (4 g/kg; 0.2 ml; i.p.). Anesthetized mice were co-injected with ^3^H-2-PAM (0.1 μCi/mouse) and ^99m^Tc-albumin (10 × 10^6^ counts/injection) in a volume of 0.1 ml in the jugular vein allowed to circulate at 1, 2, 5, 10, and 15 min. At which point, blood was collected from the descending abdominal aorta. Immediately after blood collection, transcardial perfusion with lactated Ringer’s solution was performed before collecting brain, lung, liver, and kidney. Serum and tissue samples were then gamma- and beta-radiation scintillation counted.

### Evaluation of Pralidoxime Accumulation in the Brain After NG291 Administration

CD-1 mice were subjected to 100 μg/kg NG291 i.v. or Ringer’s lactate alone (control) co-injected with ^3^H-2-PAM (0.1 μCi/injection, 1 μCi/μl) and ^99m^Tc-albumin (10 × 10^6^ counts/injection). Injectate circulated for 10 min, at which point blood was collected from the descending carotid artery. The samples were counted in a gamma counter for 3 min to determine ^99m^Tc-albumin cpm per tissue weight for the brain samples or per 20 μl in the case of injection checks and serum samples. Transcardial perfusion was done before the brain collection. The samples were then counted in a beta-scintillation counter to quantify ^3^H-2-PAM radioactivity in terms of dpm/g for brain samples or dpm/μl for injection checks and serum samples.

### Evaluating Pralidoxime and NG291 Interaction With P-Glycoprotein Transporters *via in situ* Brain Perfusion

CD-1 mice were anesthetized with 0.1–0.2 ml of 40% urethane i.p. Anesthetized mice were placed in the supine position, and the right and left jugular vein and carotid arteries were exposed. The thorax was opened from the epigastric region of the abdomen up to the sternal notch, cutting through the sternum. Jugular veins were severed bilaterally, and carotid arteries were left intact. The descending thoracic aorta was clamped with a hemostat, and a butterfly needle was introduced into the left ventricle of the heart. The syringe pump was activated, perfusing 2 ml/min for 2 min (Smith and Allen, 2003). Perfusate solution used in control groups and in NG291 studies was prepared by diluting 50 × 10^3^ dpm/ml of ^3^H-verapamil (0.09 μCi/mouse) and 10 × 10^6^ counts/ml of ^99m^Tc-albumin in Zlokovic buffer [7.19 g/L NaCl, 0.3 g/L KCl, 0.37 g/L CaCl_2_, 2.1 g/L NaHCO_3_, 0.16 g/L KH_2_PO_4_, 0.17 MgCl_2_, 0.99 g/L D-(++)-glucose, 10 g/L BSA, diluted in di H_2_O, adjusted to pH of 7.4]. In NG291 studies, 100 μg/kg of NG291 was administered i.v. 10 min prior to *in situ* brain perfusion. For pralidoxime studies, 10 μg/ml of pralidoxime (not radiolabeled, Sigma), diluted in the perfusate preparation, was used in control groups. At the end of the perfusion, the brain was collected along with perfusate samples for posterior gamma- and beta-scintillation counting.


$$\mathrm{Brain}:\mathrm{Perf}\text{usion}\left(\mu \text{l}/\text{g}\right)=\frac{\mathrm{Brain}\mathrm{dpm}}{\mathrm{Brain}\mathrm{Weight}\left(g\right)}\times \frac{\mu \text{l}\text{perfusate}}{\mathrm{dpm}\mathrm{perfusate}}$$

The equation above was used to obtain the brain to perfusion ratio for both ^99m^Tc-albumin and for ^3^H-verapamil. The ratio for ^99m^Tc-albumin was then subtracted from the ratio for ^3^H-verapamil to yield the ratio for ^3^H-verapamil taken up by the brain. The activity of the P-gp efflux system is inversely related to this delta ^3^H-verapamil ratio.

### Detecting Changes in Brain Water Content Following NG291 Administration

Sprague Dawley rats were anesthetized with a mixture of 1.5% isoflurane/70% nitrous oxide/30% oxygen. Anesthetized rats were treated i.v. with saline, 50 μg/kg NG291, or 100 μg/kg NG291 and were euthanized 24 h after the injection. The brains were harvested, and the weight was recorded. The brains were then left to dehydrate in an oven at 52°C for 72 h or until there was no change in weight after two consecutive readings. Percentage of brain water was calculated with the following equation as used by Elliott and Jasper (1949):


$$\mathrm{Brain}\mathrm{Water}\mathrm{Content}\left(\%\right)=\frac{\left(\mathrm{Wet}\mathrm{Brain}\mathrm{Weight}-\mathrm{Dry}\mathrm{Brain}\mathrm{Weight}\right)}{\mathrm{Wet}\mathrm{Brain}\mathrm{Weight}}\times 100$$

### Detecting Astrocyte Activation in NG291 Treated Rats

Sprague Dawley rats were anesthetized with a mixture of 1.5% isoflurane/70% nitrous oxide/30% oxygen. In this study, three groups were included: saline group (negative control), a group provided an acute 100 μg/kg NG291 on the first day, and a “chronic” group that was given 100 μg/kg NG291 per day for 3 days. Rats were perfused with 0.9% saline solution, and the brains were stored at −70°C on the third day after the treatment or an hour after the third dose of 100 μg/kg NG291 in the chronic group. Brains were sectioned with 20 μm thickness using a Leica CM1850 cryostat. We stained the sections with GFAP-Cy3 (Sigma C9205) and DAPI to label activated astrocytes and nuclei, respectively. Stained brain sections were observed under a Keyence BZ-X800 fluorescence microscope.

### Detecting Neurodegeneration Following NG291 Treatment

Sprague Dawley rats were anesthetized with a mixture of 1.5% isoflurane/70% nitrous oxide/30% oxygen. A group of animals was subjected to middle carotid artery occlusion (MCAO) (Rupadevi et al., 2011), which served as our positive control. In this study, three groups were included: saline group (negative control), a group provided an acute 100 μg/kg NG291 on the first day, and a “chronic” group that was given 100 μg/kg NG291 per day for 3 days. Rats were perfused with 0.9% saline solution, and the brains were stored at −70°C on the third day after the treatment or an hour after the third dose of 100 μg/kg NG291 in the chronic group. Brains were sectioned with 20 μm thickness using a Leica CM1850 cryostat. We stained the sections with the Fluoro-Jade C (FJC) Ready to Dilute Staining Protocol by Biosensis (catalog number TR-100-FJ) for non-paraffin embedded sections (Ferchmin et al., 2014; Deshpande and Phillips, 2016; Krishnan et al., 2016). Stained slides were observed under a Keyence BZ-X800 fluorescence microscope for positive FJC staining.

### Statistical Analysis

One−way analysis of variance (ANOVA) was used for analysis when the data had a normal distribution. Statistical analyses were performed using GraphPad Prism^®^ (Version 8.0.2, GraphPad Software, Inc., La Jolla, CA, United States). Bar graphs and scatterplots exhibit the mean value of the group, with error bars indicating the standard error of mean (SEM). The presented results were not tested for outliers.

## Results

### ADMET Properties of Bradykinin and NG291

ADMET profiles of bradykinin and NG291 were predicted using the pkCSM tool (see text footnote 1) as described by Pires et al. (2015) and Oladele et al. (2021). The results obtained by running the pkCSM tool for bradykinin and NG291 were summarized and compared in Table 1. Neither bradykinin nor NG291 was predicted to cross Caco-2 monolayers since highly permeable compounds should have Papp values above 8 × 10^–6^ cm/s. BK and NG291 have been predicted to have a low volume of distribution (Vd = 0.208449088; 0.086896043, respectively) in humans, unable to penetrate BBB (logBB = −2.137, −2.592, respectively) and unable to penetrate the CNS (logPS = −6.197; 6.465, respectively) (Pires et al., 2015). This can be expected as bradykinin or NG291 do not comply with Lipinski’s rule of five in terms of rotatable bonds, the number of proton donors and acceptors, and molecular weight (Lipinski et al., 2001). According to Table 1, BK and NG291 are predicted to be CYP3A4 substrates but do not play a role in its inhibition. NG291 is predicted to inhibit hERG II (Table 1), which would lead to a prolonged QT interval and potential *Torsades de pointes* in susceptible patients. Additionally, NG291 is predicted to be a P-glycoprotein efflux transporter substrate but not inhibit P-glycoprotein activity (Table 1).

### NG291 Disrupts the Blood–Brain Barrier in a Dose- and Incubation Time-Dependent Manner

**FIGURE 1:**
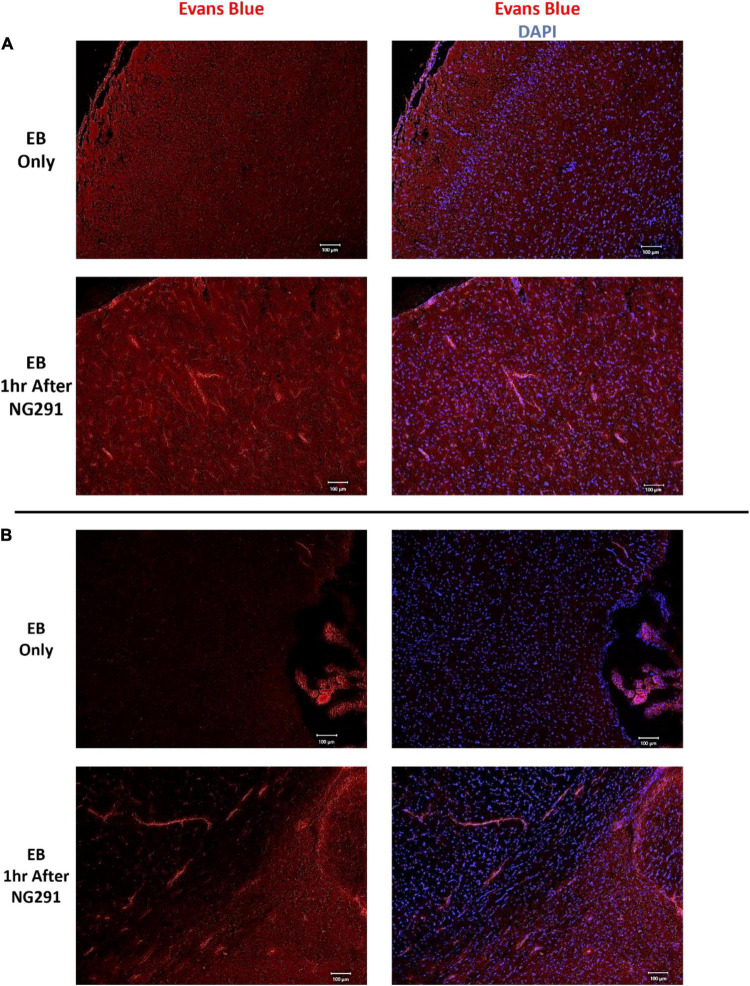
Evans blue extravasation following NG291. Sprague Dawley rats were administered 100 μg/kg of NG291 (i.v.) or saline (i.v.). Saline or NG291 was allowed to circulate for 1 h prior to Evans blue (EB, 4 ml/kg) administration. EB circulated overnight, and then the brains were harvested and coronally sectioned 20 μm thickness in a microtome. Brain sections were mounted onto slides and stained with DAPI prior to mounting onto coverslips. Evans blue extravasation was detected *via* fluorescence (excitation at 620 nm, emission at 680 nm) using a Keyence BZ-800 fluorescent microscope. Panel **(A)** includes images taken of the cortex, panel **(B)** contains images taken of brain regions adjacent to the lateral ventricles. Scale bar: 100 μm in length.

**FIGURE 2:**
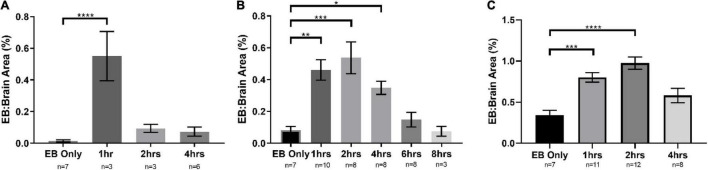
NG291 disrupts the BBB in a dose and time-dependent fashion. The area occupied by Evans blue is in proportion to the total brain area. In **(A,B)**, male Sprague Dawley (SD) rats were used. Female SD rats were used in **(C)**. SD rats were administered 50 μg/kg of NG291 (i.v.) in **(A)**. SD rats were administered 100 μg/kg of NG291 (i.v.) in **(B,C)**. Time periods in *X*-axis represent the time intervals between NG291 and Evans blue (2 ml/kg) injection. EB circulated overnight, and then the brains were harvested, sectioned, and imaged. (Data are presented as mean ± SEM). *p < 0.05, **p < 0.01, ***p < 0.001.

Several methods can be used to evaluate BBB disruption. A simple, low-cost, and fast method widely used is EB dye extravasation, which can determine BBB disruption following NG291 administration without any sophisticated equipment (Saunders et al., 2015). Here, SD rats were injected i.v. with NG291 followed by i.v. EB injection. In Figure 1, NG291 increases EB extravasation in regions surrounding blood vessels in the cortex and near lateral ventricles. To explore how long NG291 can maintain increased BBB permeability, we administered EB at various time points following an acute i.v. injection of 50 or 100 μg/kg NG291. Using this procedure, we confirmed that NG291-mediated BBB disruption is dose-dependent. Observe in Figure 2A that BBB integrity is recovered by the 2 h following 50 μg/kg of NG291 time point (0.09453 ± 0.02517; *p* = 0.7195). Doubling the concentration of NG291 (Figure 2B) causes the BBB to remain disrupted for a longer period, with detected EB extravasation returning to basal levels by the 4 h time point (0.3484 ± 0.04128; *p* = 0.0230). Figure 2C reveals that NG291 promotes similar effects with EB extravasation returning to control conditions by the 4-h time point (0.5815 ± 0.08710; *p* = 0.1102). Additionally, we noticed in Figure 2C that the BBB of female rats is more permeable to EB extravasation compared to male counterparts, as shown in Figure 2B. *Post hoc* power analysis in these studies was above 80%, with alpha = 0.05.

### NG291 Elicits Paracellular and Transendothelial Transport

**FIGURE 3:**
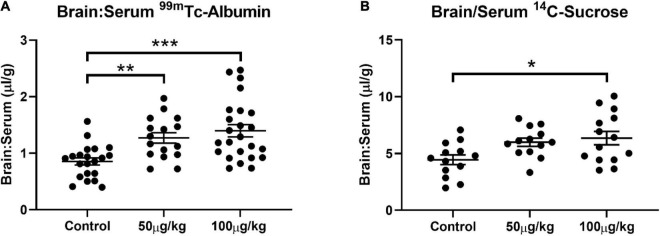
NG291 elicits paracellular and transendothelial transport. Panel **(A)** displays changes in the brain to serum ratio of ^99m^Tc-albumin 15 min after NG291 administration. Likewise, panel **(B)** is the brain to serum ratio of ^14^C-sucrose detected with and without NG291 present in the injectate. (Data is presented as mean ± SEM). *p < 0.05, **p < 0.01, ***p < 0.001.

Evans blue extravasation into the brain confirmed that NG291 transiently disrupted the BBB. For further verification of transport enhancement by NG291, we used radiolabeled probes for assessing BBB permeability. ^14^C-sucrose and ^99m^Tc-albumin are commonly used radiolabeled markers that remain in vascular compartments under normal conditions because of their incapability to effectively cross the BBB (Levin et al., 1970; Blasberg et al., 1983; Patlak et al., 1983). ^14^C-sucrose is used as the small molecular weight marker (∼342 Da) while ^99m^Tc-albumin is the high molecular weight marker (∼66.44 KDa) to probe BBB disruption (Ziylan et al., 1984; Mayhan and Heistad, 1985; Armstrong et al., 1987; Robinson and Rapoport, 1987). CD-1 mice were used in the study instead of rats to reduce excess exposure to radioactive material handled throughout the investigations. Studies had to be conducted quickly after preparing ^99m^Tc-albumin because discrepancies resulting from radioactive decay can influence the results [technetium (^99m^Tc) half-life is 6 h]. Due to the large number of studies that needed to be completed on the same day for obtaining statistically significant data, we limited the time of probe circulation to 15 min between NG291 administration and radiolabeled probe administration. As shown in Figure 3A, the brain to serum ratio of ^99m^Tc-albumin significantly increased 15 min after either 50 μg/kg (1.271 ± 0.370; *n* = 16; *p* = 0.0083) or 100 μg/kg (1.398 ± 0.533; *n* = 24; *p* = 0.0001) NG291 administration. In Figure 3B, ^14^C-sucrose accumulation was only statistically significant, compared to controls, 15 min after the injection of 100 μg/kg NG291 (6.357 ± 2.199; *n* = 14; *p* = 0.0128).

To aid in the detection of paracellular leakage, Supplementary Figure 1 shows representative images of CA1 hippocampal brain sections (interaural 5.20 mm, Bregma −3.80 mm) of SD rats treated with saline (i.v.) or 100 μg/kg of NG291. Brain sections were stained for F-actin expression (phalloidin, Alexa Fluor™ 488, green) and nuclei (DAPI, blue). F-actin expression in rat CA1 hippocampal sections was reduced in either acute or chronic NG291 administration.

### Pralidoxime Accumulation in the Central Nervous System Is Not Promoted in the Presence of NG291

**FIGURE 4:**
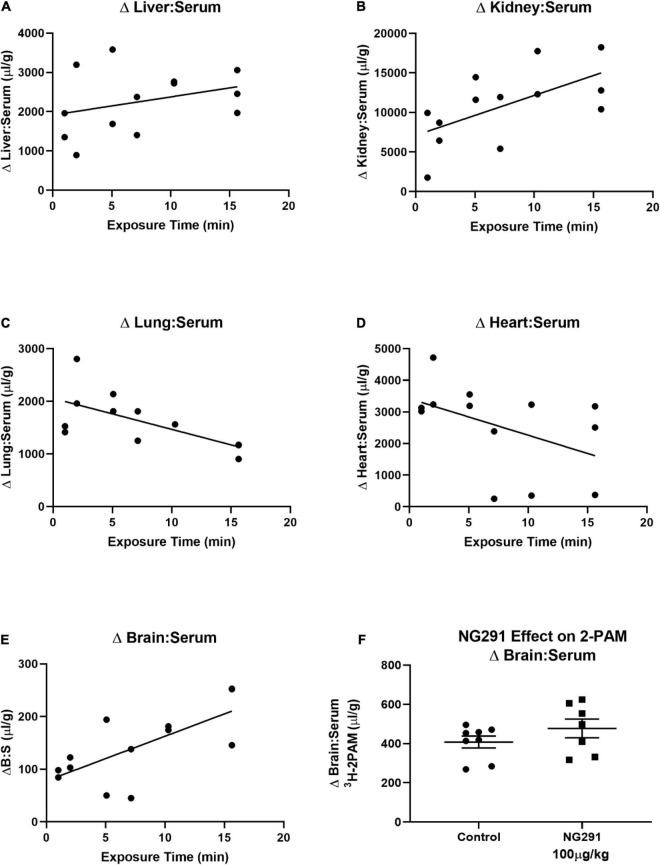
Preliminary 2-PAM kinetics and the effect of NG291 on the 2-PAM brain to serum ratio. Preliminary ^3^H-2-PAM kinetics was evaluated in tissue, and serum samples of mice were collected 1–15 min following ^3^H-2-PAM injections **(A–E)**. In **(F)**, 100 μg/kg of NG291 was administered along with ^3^H-2-PAM and ^99m^Tc-albumin and allowed to circulate for 15 min prior to harvesting brain and serum. Unpaired *t*-test reveals a *p*-value of 0.23 when comparing the ^3^H-2-PAM brain to serum ratios between control and NG291 conditions **(F)**. In **(F)**, data is presented in terms of mean ± SEM.

Pralidoxime is an FDA-approved medication used to recover acetylcholinesterase activity before covalently binding to organophosphates. Such effects are induced by some insecticides or nerve agents employed in warfare (Cannard, 2006; Buckley et al., 2011; Ferchmin et al., 2015). The inability of 2-PAM to recover acetylcholinesterase activity in the CNS has restricted the overall success of the drug (Okuno et al., 2008). This is because 2-PAM is unable to reach therapeutically relevant concentrations in the CNS (Sakurada et al., 2003, 2015). 2-PAM is a small positively charged drug that should have similar kinetics as the ^14^C-sucrose marker used in Figure 3B. In Figure 3B, the 50 μg/kg dose of NG291 was unable to significantly increase ^14^C-sucrose accumulation in the brain. Following suit, we opted to use the higher dose (100 μg/kg) of NG291 to see if we can achieve a significant increase in 2-PAM concentrations in the brain. We first determined the baseline pharmacokinetics of ^3^H-2-PAM in the liver, kidney, lung, heart, and brain in proportion to serum (Figures 4A–E). Longer exposure times lead to the accumulation of ^3^H-2-PAM in the liver and kidney (Figures 4A,B), whereas lung and heart tissue results in a reduction in ^3^H-2-PAM (Figures 4C,D). In Figure 4E, we observe signs of ^3^H-2-PAM accumulation in the brain with longer exposure times. With this observation, we sought out to determine if NG291 mediated increase in BBB permeability facilitates ^3^H-2-PAM penetration in the CNS. However, there was no significantly increased accumulation of ^3^H-2-PAM (482.5 ± 127.3 μl/g; *p* = 0.2269) in CD-1 mice treated with ^3^H-2-PAM and 100 μg/kg NG291 (Figure 4F).

### NG291 and Pralidoxime Interactions With P-Glycoprotein Transporters *via* ^3^H-Verapamil Accumulation

**FIGURE 5:**
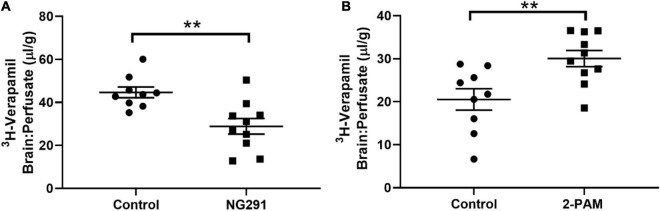
Evaluating NG291 and 2-PAM interaction with P-gp transport. P-gp transport potential was evaluated by quantifying ^3^H-verapamil accumulation in the brain in proportion to that in the perfusate. Unpaired *t*-test reveals *p* = 0.0028 in **(A)** and *p* = 0.0067 for **(B)**. Data are presented as mean ± SEM (control *n* = 9, NG291 *n* = 10, and 2-PAM *n* = 10). *Post hoc* power analysis was calculated with an alpha of 0.05 at 94.6% for **(A)** and 86.1% for **(B)**. **p < 0.01.

Verapamil is a P-gp substrate. Increased ^3^H-verapamil concentrations in the brain indicate the presence of additional substrates interacting with P-gp transporters. Decreased ^3^H-verapamil concentrations in the brain indicate enhanced P-gp transporter activity. No changes in Verapamil concentrations indicate the absence of interactions with P-gp transporters. NG291 induced P-gp transporter activity as it significantly decreased ^3^H-verapamil accumulation in the brain (Figure 5A, *p* = 0.0028), suggesting that NG291 plays a role in increased P-gp transport activity. However, 2-PAM displaced ^3^H-verapamil from binding to P-gp transporters (Figure 5B, *p* = 0.0067), suggesting that 2-PAM is a P-gp substrate. This may also explain why there was no increased 2-PAM entry into the brain when NG291 induced BBB leakage.

### NG291 Does Not Change the Brain Water Content in Either Male or Female Rats

**FIGURE 6:**
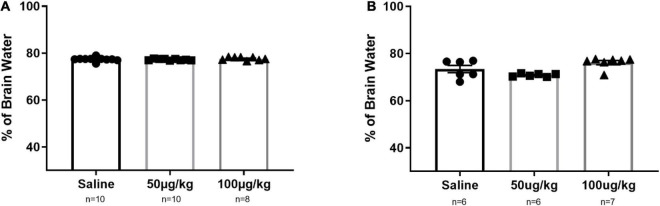
NG291 influence on the brain water content in male and female Sprague Dawley rats. Brains were harvested and weighed after being left overnight following the administration of the experimental condition. Then, they were dehydrated until the weight no longer fluctuated. Panel **(A)** represents data for male rats, while female rats are represented in **(B)**. Data are presented as mean ± SEM. One-way ANOVA with *post hoc* Tukey’s test for multiple comparisons was employed (mean ± SEM; *p* > 0.05 in all groups).

One of the main risks with BBB disruption has been associated with changes in the brain water content (Abbott, 2000; Marceau et al., 2020). The principle is that a compromised BBB allows access to low and high molecular weight molecules would result in vasogenic edema. In this study, we quantify brain water content to ensure that the risks associated with this proposed drug delivery strategy do not outweigh the potential benefits. The data were obtained by following an established protocol to determine the brain water content (Mao et al., 2015). As shown in Figures 6A,B, there is no statistically significant change in brain water content in male and female SD rats treated with NG291.

### Acute or Chronic NG291 Administration Is Not Associated With Astrocyte Activation

**FIGURE 7:**
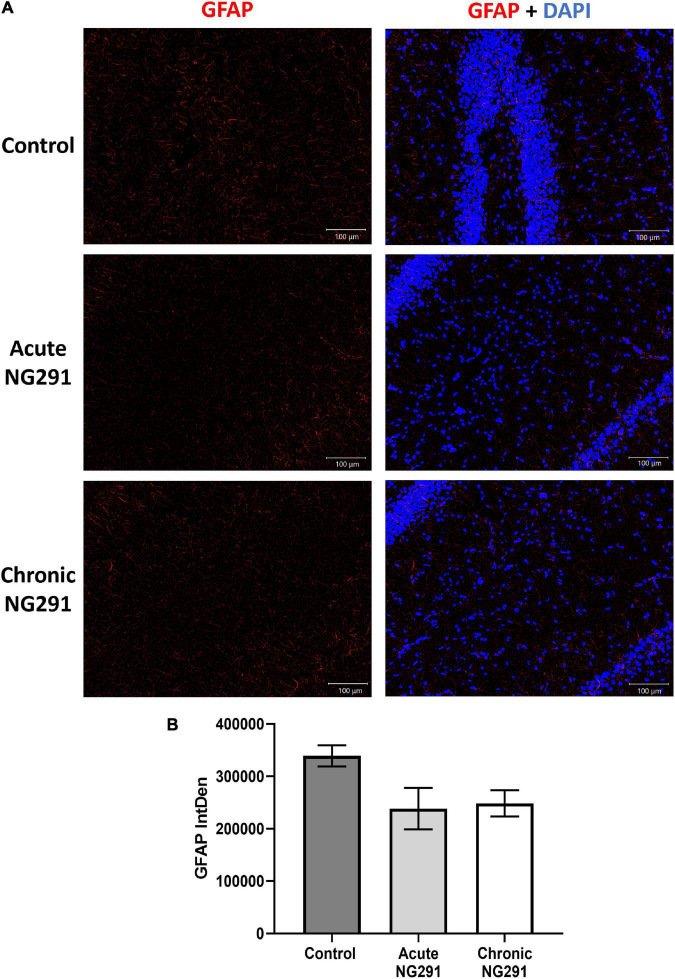
Astrocyte activation: analysis *via* GFAP detection. Sprague Dawley rats were treated with: saline, acute (100 μg/kg) NG291, or chronic NG291 (100 μg/kg every 24 h for 3 days, total three doses). Harvested brains were sectioned coronally 20 μm thick. Keyence BZ-X800 fluorescence microscope was used to detect positive GFAP cell staining. Brain sections (interaural 5.20 mm, Bregma –3.80 mm) were imaged at 10× under the same conditions. Panel **(A)** displays GFAP expression at the CA1 hippocampus in different experimental conditions. The scale bar: 100 μm in length. Panel **(B)** shows GFAP quantification across the entire cross-section by stitching 10× images together (200–210 images) using the software supplied by the Keyence BZ-X800 fluorescence microscope. Data are expressed in terms of mean ± SEM. Ordinary one-way ANOVA was conducted, revealing *p*-values above 0.05 when comparing GFAP expression in control conditions vs. acute or chronic 100 μg/kg of NG291 treated rats. *Post hoc* power analysis was calculated with an alpha of 0.05 at 80.6%. *n* = 5 in all conditions.

In order to test the hypothesis (Siracusa et al., 2019), SD rats were treated with: saline; acute 100 μg/kg NG291 treatment as well as 100 μg/kg of NG291 administered every 24 h for 3 days (chronic). Following imaging of brain sections (interaural 5.20 mm, Bregma −3.80 mm) in Figure 7A, GFAP-Cy3 positive activated astrocytes in CA1 hippocampus in the different experimental conditions are presented. In Figure 7B, GFAP-Cy3 fluorescent intensity was calculated *via* ImageJ throughout a brain cross-section (interaural 5.20 mm, Bregma −3.80 mm). One-way ANOVA revealed *p*-values above 0.05 when comparing GFAP expression in control conditions with that of acute or chronic 100 μg/kg NG291-treated rats. *Post hoc* power analysis was calculated with an alpha of 0.05 at 80.6%. *n* = 5 in all conditions.

### NG291 Administration Did Not Contribute to Neurodegeneration

**FIGURE 8:**
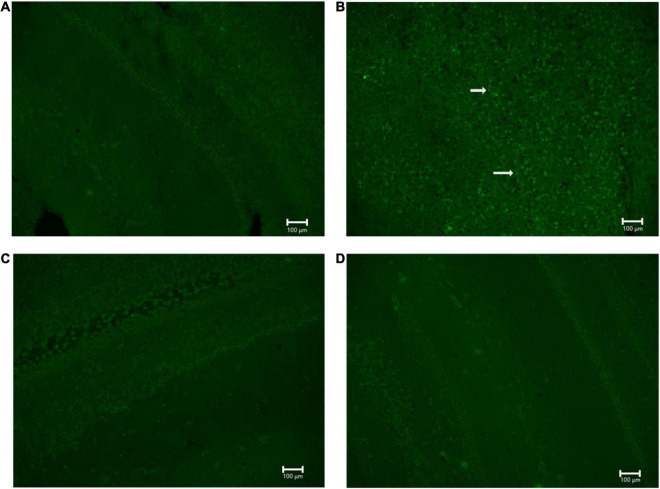
Analysis of neurodegeneration using Fluoro-Jade C. Sprague Dawley rats underwent MCAO procedure and the posteromedial cortical amygdala examined for Fluro-Jade C positive cells **(A)** no MCAO + saline (control); **(B)** MCAO + saline (positive control); **(C)** MCAO + 100 μg/kg NG291; **(D)** MCAO + 100 μg/kg of NG291 every 24 h for 3 days. Transcardial perfusion was performed on the third day after the initial treatment for conditions **(A–C)**. Animals in condition **(D)** were perfused 1 h after the third NG291 dose. Brains were sectioned coronally with 20 μm of thickness. Keyence BZ-X800 fluorescence microscope was used to detect positive Fluoro-Jade C cell staining. Panels **(A–D)** were imaged at 10× under the same conditions. White arrows indicate positive staining for Fluoro-Jade C. Scale bar = 100 μm.

For NG291 mediated BBB disruption to represent a viable strategy to facilitate therapeutic delivery to the brain, the benefits must outweigh the risks. FJC staining was employed to detect signs of neurodegeneration following NG291 treatment. FJC stains all degenerating neurons, regardless of specific insult or mechanism of cell death (Schmued et al., 2005; Li et al., 2011). Brain sections of saline-treated rats are presented in Figure 8A. As a positive control, rats underwent an MCAO procedure to provide significant FJC positive cell staining for neurodegeneration (Figure 8B). Positive FJC staining was absent in rats treated with either a single 100 μg/kg NG291 dose 3 days before harvesting the brain (Figure 8C) or in rats treated daily with 100 μg/kg NG291 for 3 days (Figure 8D).

## Discussion

The development of therapeutics that can effectively treat CNS pathologies is challenging because a vast majority of the tested drugs do not cross the BBB (Begley, 2004; Henderson and Piquette-Miller, 2015). Developing an efficient adjunct therapy or drug modification that can improve a specific drug to reach therapeutic concentrations in the CNS is highly rare (Su and Sinko, 2006; DiMasi, 2014a,b). Medicinal chemistry can be employed to make medicines more lipid-soluble or to conjugate the compound to another compound that takes advantage of endogenous transport mechanisms to increase therapeutic penetration across the BBB (Tarragó-Trani and Storrie, 2007; Reichel, 2009). However, these strategies negatively impact EC50 values as the therapy tends to have larger volumes of distribution and lower binding affinity toward the intended target (Borlongan and Emerich, 2003; Pardridge, 2005; Henderson and Piquette-Miller, 2015; Dong, 2018). On the other hand, to avoid costly modifications of the chemical structure of the therapeutic, another strategy seeks to disrupt BBB integrity to facilitate drug delivery (Li et al., 2019). Several techniques were developed in the past, such as the use of ultrasound, hyperosmotic solutions, and the use of antidepressants (for review, see Pardridge, 2012). Due to the nature of such a strategy, BBB disruption has been employed in emergencies where the benefits outweigh the risks (Li et al., 2019). In this work, we study the action of a stable BK analog called NG291. This modified peptide has high selectivity toward the BKB2R and can induce a rapid and transient increase in BBB permeability.

Pharmacokinetic properties of NG291 and BK have been outlined in Table 1, along with toxicological parameters that need to be considered. The pkCSM software predicts that BK or NG291 are incapable of passively crossing the BBB since their chemical properties do not meet the standards described in Lipinski’s rule of five (Lipinski et al., 2001). The software also predicts that NG291 may inhibit hERG II potassium channels. Inhibition of hERG potassium channels may prolong the QT segment that results in *Torsades de pointes*-susceptible patients, resulting in syncope and sudden death (Jing et al., 2015; Kenakin, 2016). While further investigations are necessary, our results provide valuable information when considering candidates for coupling to an NG291 adjuvant therapy, for instance, to avoid more than one drug at a time that may inhibit hERG (van Noord et al., 2011; Jing et al., 2015; Klein et al., 2016).

Evans blue extravasation in females was noticeably higher in all conditions, including the control group. The literature suggests that elevated female serum gonadotropins are associated with decreased expression of gap junction proteins such as connexin-43 that leads to increased BBB permeability observed in female patients (Wilson et al., 2008). This association between elevated serum gonadotropins and increased BBB permeability has been suggested to be a contributing factor for the high incidence of stroke and Alzheimer’s disease in reproductively senescent women (Wilson et al., 2008; Brzica et al., 2018).

Using radiolabeled probes of different molecular weights, we determined if NG291 increased BBB permeability by changing paracellular and/or transcellular transport mechanisms. The mechanism is of the utmost importance to understand what type of molecule will benefit the most from previously described BKB2R mediated disengagement of BBB tight junctions (Sanovich et al., 1995; Elliott et al., 1996; Borlongan and Emerich, 2003; Savard et al., 2013). Selective BKB2R signaling has been linked in the literature to increased paracellular transport across the BBB based on electron microscopy images following lanthanum (electron-dense 139 Da probe) leakage through tight junctions (Sanovich et al., 1995; Emerich et al., 2001; Borlongan and Emerich, 2003; Saaber et al., 2014). However, lanthanum has been shown to be able to inhibit intracellular calcium mobilization (Korc and Schöni, 1987; Jan et al., 1998). The utilization of lanthanum might have reduced the detection of calcium-dependent transcellular transport mechanisms mediated by BKB2R signaling while preserving signaling pathways that trigger tight junction disengagement. Despite the literature associating BKB2R stimulation to paracellular disruption, our results suggest a more complex picture with both 50 μg/kg and 100 μg/kg NG291 enhancing the delivery of large molecules that traditionally rely on vesicular transport (as seen with ^99m^Tc-albumin accumulation) (Mehta and Malik, 2006; Blyth et al., 2009; Fung et al., 2018). Furthermore, an increase in paracellular transport was detected with higher concentrations of NG291. It is not impossible for albumin and other large molecules in serum to cross the BBB *via* paracellular mechanisms, and there is nothing stopping sucrose from being shuttled *via* a vesicle. However, should BKB2R mediated increases in BBB permeability be caused solely by paracellular leakage, ^14^C-sucrose would likely be favored since it is smaller. Instead, we see a statistically significant accumulation of ^99m^Tc-albumin with 50 μg/kg of NG291 and a statistically significant accumulation of ^99m^Tc-albumin and ^14^C-sucrose with 100 μg/kg of NG291. NG291-induced transcellular transport is likely mediated by absorptive or “Trojan Horse” style receptor-mediated transcytosis due to selective albumin delivery detected in the brain with smaller NG291 concentrations. Increased incidence of transcytosis would likely explain why significant sucrose accumulation is detected with higher concentrations of NG291 administered.

Rats treated with 100 μg/kg of NG291 (acute or chronically) show reduced Phalloidin-488 fluorescent intensity in CA1 hippocampal sections suggesting that NG291 may increase paracellular transport through the disengagement of tight junction components in brain endothelial cells. F-actin, claudin-5, ZO-1, and ZO-2 are found in functional tight junction complexes between BBB endothelial cells (Stamatovic et al., 2016). Decreased F-actin expression has been associated with tight junction disengagement and reduced ZO-1 and claudin-5 expression (Liu et al., 2008; Stamatovic et al., 2016). This is likely explained by BKB2R-mediated vasodilator-stimulated phosphoprotein (VASP) activation. Signaling pathways associated with [Ca^2+^]_*i*_, PI3K/Akt, and NO/cGMP are activated by BKB2R stimulation in brain endothelial cells (Comerford et al., 2002; Fischer et al., 2004). [Ca^2+^]_*i*_, PI3K/Akt, and NO/cGMP signaling lead to protein kinase G activation, which activates VASP (Comerford et al., 2002; Fischer et al., 2004). VASP colocalizes with ZO-1, and its activation has been associated with decreased F-actin expression, tight junction disengagement, and increased BBB permeability (Comerford et al., 2002; Fischer et al., 2004; Davis et al., 2010).

Pralidoxime is one of the suggested therapies to be administered to patients exposed to organophosphate agents such as insecticides (such as parathion) and nerve agents (such as sarin gas) to recover acetylcholinesterase before being covalently bound to the organophosphate (Szinicz et al., 2007; Seaman, 2008; Colovic et al., 2013). Tissue and serum samples were collected at several time points following ^3^H-2-PAM administration. These data confirm that ^3^H-2-PAM is quickly eliminated as it accumulates in the kidney and the liver while concentrations in the lung and heart decrease with time. While the literature supports that 2-PAM is unable to reach therapeutically relevant concentrations in the CNS (Buckley et al., 2011; Torres-Rivera et al., 2013; Ferchmin et al., 2015), our results are in agreement that ^3^H-2-PAM has some degree of brain penetration (Sakurada et al., 2003).

The BBB prevents more than 90% of all small-molecule drugs and almost all larger therapeutics from entry into the brain (Pardridge, 2005). Many drugs that are predicted to cross the BBB show very low brain permeation because they are substrates of efflux transporters, such as P-gp efflux transporters (Hersh et al., 2016). P-gp is a member of the ATP binding cassette family that actively transports a wide range of compounds (roughly one-third of all marketed drugs) out of the brain and represents an additional challenge when designing CNS therapeutics (Demeule et al., 2002; Su and Sinko, 2006; Miller et al., 2008). In Table 1 NG291 was predicted to interact with P-gp but not as an inhibitor. To uncover how NG291 interacts with P-gp, we utilized tritiated verapamil accumulation as a marker for P-gp transport activity. Verapamil is a known P-glycoprotein substrate, and its entry into the brain is inversely related to P-gp activity at the BBB (Summers et al., 2004; Anekonda et al., 2011). While 2-PAM is reported to lack affinity toward P-gp *in vitro* (Gallagher et al., 2016), we demonstrated (*via in situ* brain perfusions in mice) that 2-PAM does interact with P-gp. The discrepancy between the results is difficult to compare, considering the different models used (*in vitro* vs. *in vivo*).

Ideally, therapeutic candidates coupled to NG291 adjuvant therapy should not be P-gp substrates for optimal performance. Likewise, NG291 may have additional applications in the treatment of Alzheimer’s disease. Reduced P-gp activity has been detected in Alzheimer’s disease patient’s BBB, contributing to the accumulation of amyloid beta-peptide and plaque formation observed in Alzheimer’s disease (Banks, 2012). Female patients appear to have reduced P-gp expression in the brain (Van Assema et al., 2012), which could be why greater overall leakiness to EB was detected in female SD rats (Cho et al., 2016). The ability to enhance P-gp activity to remove amyloid-β from the CNS should be studied.

So far, we have evaluated the therapeutic potential of NG291. However, a clear understanding of the risks associated with this therapeutic strategy is imperative. Earlier, we have identified the possibility of hERG inhibition which needs to be evaluated. Epileptic patients should avoid NG291 treatment due to up-regulated P-gp activity. Most anti-seizure medications are P-gp substrates, and increased P-gp activity would make it very difficult to counteract status epilepticus with medications (Banks, 2016). As previously mentioned, the main concerns of achieving BBB disruption *via* bradykinin receptor agonism involve neuroinflammation and vasogenic edema (Pangalos et al., 2007; Sandoval and Witt, 2008; Muradashvili et al., 2012). We showed that NG291 does not increase the brain water content in male or female rats. This result suggests that NG291-mediated BBB disruption is subtle compared to the results obtained with hyperosmotic mannitol injections (Jiang et al., 2012; Weidman et al., 2016). Vasogenic edema is not achieved by NG291-mediated BBB disruption. To ensure that NG291 does not contribute to astrocyte activation, we stained for activated astrocytes (GFAP-Cy3) and nuclei (DAPI) in rat brain CA1 hippocampal sections. Anti-GFAP immunofluorescence staining revealed that NG291 did not significantly modulate astrocyte activation. Besides vasogenic edema and neuroinflammation, increased BBB permeability is associated with neurodegeneration. FJC staining was utilized to detect signs of NG291-mediated neurodegeneration, regardless of apoptosis or necrosis. No signs of neurodegeneration were observed in rat brains harvested on the third day after a single NG291 dose or 1 h after the third dose of NG291 administered once per day for 3 days. NG291 did not elicit the common inflammation associated with BBB disruption, as observed in various studies (Sandoval and Witt, 2008; Jiang et al., 2012; Chen et al., 2016). Taking these results together, NG291 is capable of increasing BBB permeability without contributing to the risks associated with BBB disruption.

Despite these observations, a fundamental understanding of the biochemical pathways recruited by NG291 to incite BBB disruption is not clear. Therefore, additional studies are required to elucidate the signaling events triggered within the endothelial cells and how the NVU responds. In addition, while NG291 was detected to incite BBB disruption, further studies are needed to confirm if there is preferential disruption of paracellular or transcellular routes of transport. Understanding the transportation routes enhanced will assist in the search for CNS-restricted therapeutics that could benefit from NG291 adjuvant therapy. Finally, additional studies are also necessary to study the effects of BKB2R activation peripherally and evaluate additional risks associated with the use of NG291. These studies will allow us to gauge further the risk-benefit ratio associated with the use of NG291.

## Author’s Note

This work exposes the gaps in understanding how bradykinin B2 receptor stimulation by NG291 increases blood–brain barrier permeability. We show here that protease-resistant NG291 transiently increases BBB permeability. Contrary to the literature, BKB2R stimulation may enhance transcellular transport. Also, our results suggest an increase in P-gp activity after treatment of NG291 in mice. Further investigations on the therapeutic applications of BKB2R agonists may be of value for Alzheimer’s disease patients being given antibody therapies that target amyloid-beta. NG291 could facilitate the delivery of antibody therapies across the BBB while fomenting efflux of amyloid beta through its ability to increase P-gp efflux transport activity. NG291 is capable of increasing BBB permeability without influencing brain water content, astrocyte activation, or neurodegeneration.

## Data Availability Statement

The raw data supporting the conclusions of this article will be made available by the authors, without undue reservation.

## Ethics Statement

This study was reviewed and approved by Universidad Central del Caribe or by the Veterans Affairs Puget Sound Health Care System’s Institutional Animal Care and Use Committee. All animals were housed and handled following its protocols.

## Author Contributions

SR-M performed the studies, statistical analysis, and wrote the first draft of the manuscript. AM provided a conceptualization and design of the experiments, funding, guided the investigation, and analyzed the results. WB and ME provided the protocols, equipment, and raw materials for all studies that utilized radiolabeled probes. HU contributed to the conceptualization of this work by providing his expertise in kinin pharmacology. All authors contributed to manuscript revision, read, and approved the submitted version.

## Author Disclaimer

All claims expressed in this article are solely those of the authors and do not necessarily represent those of their affiliated organizations or those of the publisher, the editors, and the reviewers. Any product that may be evaluated in this article or claim that may be made by its manufacturer is not guaranteed or endorsed by the publisher.

## Conflict of Interest

The authors declare that the research was conducted in the absence of any commercial or financial relationships that could be construed as a potential conflict of interest.

## Publisher’s Note

All claims expressed in this article are solely those of the authors and do not necessarily represent those of their affiliated organizations, or those of the publisher, the editors and the reviewers. Any product that may be evaluated in this article, or claim that may be made by its manufacturer, is not guaranteed or endorsed by the publisher.

## Footnotes

1
http://biosig.unimelb.edu.au/pkcsm/prediction

2
https://jsme-editor.github.io/dist/JSME_test.html

3
https://www.medilumine.com/product/bradykinin-b2-receptor-agonist/

4
http://imagej.nih.gov/ij
