# Supplementary material for: The Bradykinin B2 Receptor Agonist (NG291) Causes Rapid Onset of Transient Blood–Brain Barrier Disruption Without Evidence of Early Brain Injury
Source: Front Neurosci. 2021 Dec 15;15:791709. doi: 10.3389/fnins.2021.791709 (PMC8715084; doi:10.3389/fnins.2021.791709)
Supplement: Supplementary file 1 [file Image_1.pdf]

Supplementary Material:

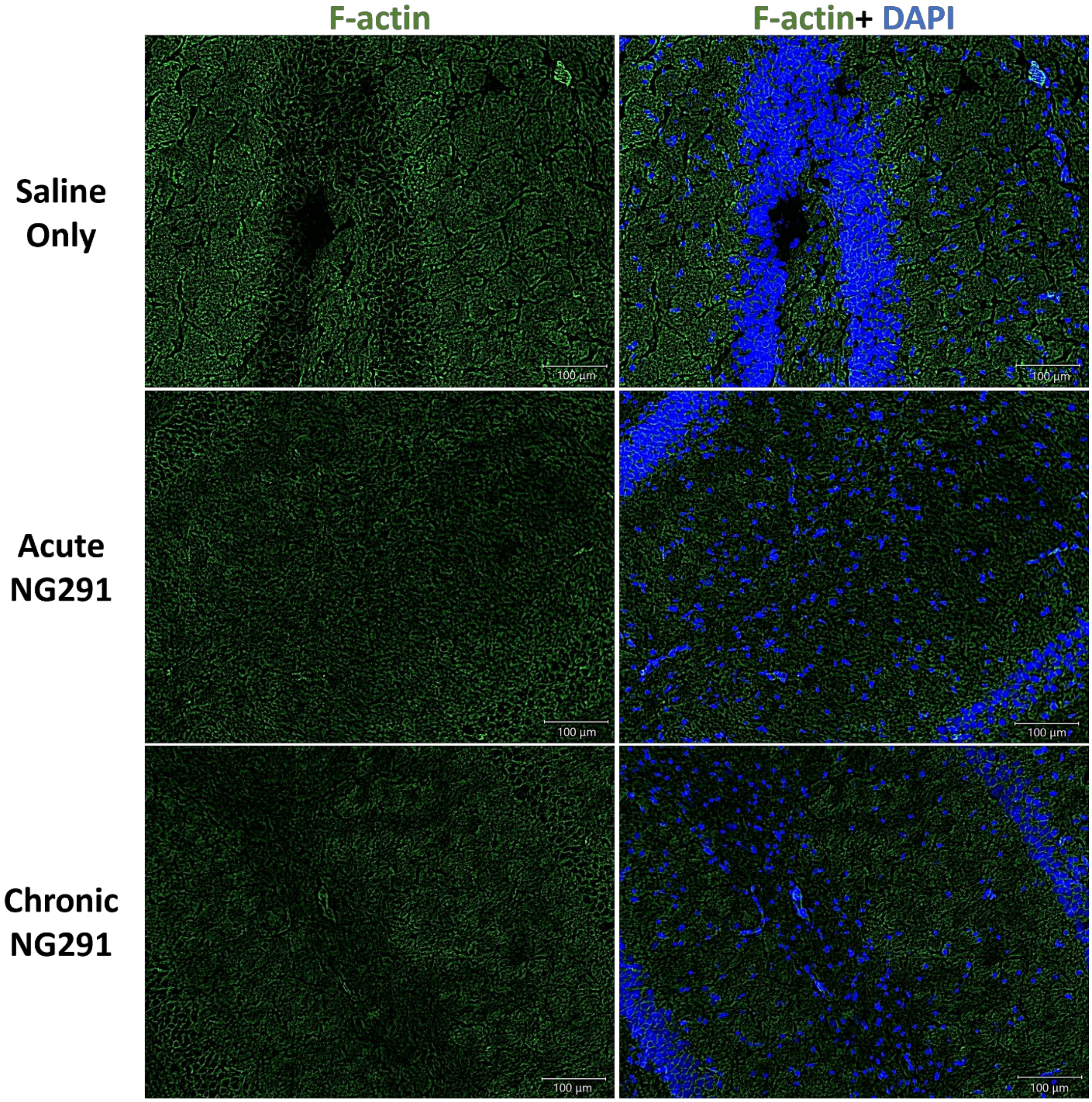

Figure SM1: NG291 plays a role in reducing the expression of F-actin in rat brain hippocampus. Sprague Dawley rats were treated with saline (control), Acute NG291 (100µg/kg NG291 three days prior to saline perfusion), and chronic NG291 (single 100µg/kg NG291 dose per day for three days with brain perfusion conducted 1hr after the final dose) were sectioned 20µm thick. Brain sections were stained for F-actin (phalloidin, Alexa Fluor™ 488, green) and nuclei (DAPI, blue). Figure SM1 shows decreased F-actin immunofluorescence when exposed to NG291. Scale bar= 100µm.
